# Discordant Spirometry and Impulse Oscillometry Assessments in the Diagnosis of Small Airway Dysfunction

**DOI:** 10.3389/fphys.2022.892448

**Published:** 2022-06-22

**Authors:** Lifei Lu, Jieqi Peng, Ningning Zhao, Fan Wu, Heshen Tian, Huajing Yang, Zhishan Deng, Zihui Wang, Shan Xiao, Xiang Wen, Youlan Zheng, Cuiqiong Dai, Xiaohui Wu, Kunning Zhou, Pixin Ran, Yumin Zhou

**Affiliations:** ^1^ National Center for Respiratory Medicine, State Key Laboratory of Respiratory Disease, National Clinical Research Center for Respiratory Disease, Guangzhou Institute of Respiratory Health, The First Affiliated Hospital of Guangzhou Medical University, Guangzhou, China; ^2^ Guangzhou Laboratory, Guangzhou, China

**Keywords:** spirometry, impulse oscillometry, small airway dysfunction, COPD, computed tomography

## Abstract

**Background and objective:** Spirometry is commonly used to assess small airway dysfunction (SAD). Impulse oscillometry (IOS) can complement spirometry. However, discordant spirometry and IOS in the diagnosis of SAD were not uncommon. We examined the association between spirometry and IOS within a large cohort of subjects to identify variables that may explain discordant spirometry and IOS findings.

**Methods:** 1,836 subjects from the ECOPD cohort underwent questionnaires, symptom scores, spirometry, and IOS, and 1,318 subjects were examined by CT. We assessed SAD with R_5_-R_20_ > the upper limit of normal (ULN) by IOS and two of the three spirometry indexes (maximal mid-expiratory flow (MMEF), forced expiratory flow (FEF)_50%_, and FEF_75%_) < 65% predicted. Multivariate regression analysis was used to analyze factors associated with SAD diagnosed by only spirometry but not IOS (spirometry-only SAD) and only IOS but not spirometry (IOS-only SAD), and line regression was used to assess CT imaging differences.

**Results:** There was a slight agreement between spirometry and IOS in the diagnosis of SAD (kappa 0.322, *p* < 0.001). Smoking status, phlegm, drug treatment, and family history of respiratory disease were factors leading to spirometry-only SAD. Spirometry-only SAD had more severe emphysema and gas-trapping than IOS-only SAD in abnormal lung function. However, in normal lung function subjects, there was no statistical difference in emphysema and gas-trapping between discordant groups. The number of IOS-only SAD was nearly twice than that of spirometry.

**Conclusion:** IOS may be more sensitive than spirometry in the diagnosis of SAD in normal lung function subjects. But in patients with abnormal lung function, spirometry may be more sensitive than IOS to detect SAD patients with clinical symptoms and CT lesions.

## Introduction

A small airway is usually defined as the inner diameter of the broncho less than 2 mm without cartilage (Macklem, 1998) and is considered to be the main site of airflow resistance in obstructive lung disease (Burgel, 2011; Gove et al., 2018). It is also referred to as the “silent zone” because it contributes relatively little to overall airway resistance and is difficult to detect (McNulty and Usmani, 2014). Characteristics of small airway obstruction included premature airway closure, gas trapping, regional heterogeneity, and exaggerated volume dependence of airflow limitation (Konstantinos Katsoulis et al., 2016). In recent years, various lung function tests specifically have been developed based on small airway physiology in order to detect small airway pathologies earlier and to provide earlier diagnosis and intervention. Currently, spirometry is commonly used in clinical practice to assess small airway dysfunction (SAD), and the advent of impulse oscillometry (IOS) can complement spirometry. We adopted the IOS index to analyze the difference between 5 HZ and 20 HZ of reactance (R_5_-R_20_) > the upper limit of normal (ULN) (Liang et al., 2021) and two of the three spirometry indexes (maximal mid-expiratory flow (MMEF) and forced expiratory flow (FEF)_50%_, and FEF_75%_) < 65% predicted (Xiao et al., 2020) to diagnose SAD. However, we found that it was not uncommon to encounter normal spirometry but SAD on IOS. This phenomenon has been described as the inconsistency between spirometry and IOS in the diagnosis of SAD.

This study aimed to evaluate the association between spirometry and IOS to assess SAD in a large sample. In addition, our goal was to identify relevant factors that may explain the inconsistency between spirometry and IOS. Finally, we analyzed the differences in CT imaging (emphysema and gas trapping) between the two discordant groups in different subjects.

## Methods

### Study Design and Participants

This was a prospective observational cohort study (ECOPD cohort) in Guangdong (Wu et al., 2021). The subjects were continuously recruited and tested in Wengyuan,Lianping countryside and Guangzhou city, Guangdong Province from July 2019 to August 2021.

The subjects in this study must be 40–80 years old and undergo lung function tests, IOS, radiological imaging, and epidemiological investigation of chronic obstructive pulmonary disease. The subjects would be excluded in the following criteria: (I) aged <40 years or >80 years; (II) respiratory infection or exacerbation in the four weeks prior to screening; (III) heart attack (myocardial infarction, malignant arrhythmia) in the past three months; (IV) hospitalized for heart disease within the past one month, (V) chest, abdomen, or eye surgery in the past three months, (VI) previous lobectomy; (VII) malignant tumors newly discovered and being treated; (VIII) receiving anti-*tuberculosis* drug treatment or active pulmonary tuberculosis; (IX) history of mental disorders, auditory hallucinations, visual hallucinations or taking antipsychotic drugs; (X) history of cognitive disorders, including dementia or cognitive disorders; (XI) history of high paraplegia; and (XII) pregnant or lactating women.

Basic demographic variables included gender, age and body mass index (BMI), smoking index, smoking status, family history of respiratory disease, drug treatment, modified Medical Research Council (mMRC) dyspnoea scale score and COPD assessment test (CAT) score. Chronic obstructive pulmonary disease was diagnosed based on spirometry (criterion for airflow limitation as a post-bronchodilator fixed ratio of FEV_1_/FVC <0.70), normal lung function was defined as FEV_1_/FVC ≥0.70 and FEV_1_ predicted % ≥ 80% after bronchodilation, and abnormal lung function was defined as either FEV_1_ predicted % < 80% or FEV_1_/FVC <0.70. SAD was assessed by the IOS index using R_5_–R_20_ greater than the upper limit of normal (ULN), which was calculated based on a multicenter, Chinese healthy population impulse oscillometry study (Liang et al., 2021). SAD was diagnosed by spirometry based on at least two of the following three indexes (MMEF, FEF_50%_, and FEF_75%_), less than 65% predicted, and the predicted values were based on a multicenter study of spirometry reference values in a healthy Chinese population (Jian et al., 2017). All subjects were divided into four groups based on lung function and IOS criteria for assessing SAD: 1) SAD assessed by both spirometry and IOS (concordant SAD); 2) SAD assessed by neither spirometry nor IOS (concordant NO SAD); 3) SAD diagnosed by only spirometry but not IOS (spirometry-only SAD); 4) SAD diagnosed by only IOS but not spirometry (IOS-only SAD).

The Ethics Committee of the First Affiliated Hospital of Guangzhou Medical University approved this study protocol (2018–53). Written informed consent was obtained from all subjects prior to inclusion.

### Impulse Oscillometry

The respiratory resistance and reactance were measured by IOS (MasterScreen IOS, Hochberg Germany). Subjects never received any medication before the IOS test. The operator gently pressed the subject’s cheeks with both hands to avoid cheek vibration affecting the accuracy of the measurement (King et al., 2020). Respiratory system impedance (Z_rs_) consists of resistance (R_rs_) and reactance (X_rs_). R_rs_ at 5 Hz (R_5_) indicates total respiratory resistance and R_rs_ at 20 Hz (R_20_) represents central airway resistance; R_5_–R_20_, an index of SAD, reflects peripheral airway resistance; X_5_, reactance value at 5Hz, is a measure of the stiffness of the entire system. Resonance frequency (F_res_), where E_rs_ and I_rs_ make equal and opposite contributions to impedance and reactance, is a sensitive index reflecting increased resistance; AX, the area under X_5_ and F_res_, is considered to be an important index of early detection and prognosis of COPD (Lipworth and Jabbal, 2018), reflecting the comprehensive index of reactance.

### Lung Function Test

According to the ATS/ERS spirometry guidelines (Miller et al., 2005), the acceptable standard of repeatability was a single test that included no hesitation in the onset of expiration and extrapolation volume <5% FVC or 0.15L. The difference between the two largest values of FEV_1_ and FVC was within 0.15 L. Subjects were required to be measured at least three times to ensure reproducibility. For the bronchodilator test, subjects inhaled 400 µg of salbutamol through a nebulizer canister, and a pulmonary function test was performed after 20 min.

### Computed Tomography

Percent emphysema was defined as the total percentage of both lungs with attenuation values less than -950 Hounsfield units on inspiratory images, and percent gas trapping was defined as the total percentage of both lungs with attenuation values less than -856 Hounsfield units on expiratory images (Kim et al., 2014; Lynch et al., 2015). The results of the data were evaluated by at least two clinical radiologists.

### Statistical Analysis

Statistical analysis was performed with SPSS statistics version 26.0 (IBM Corp. Armonk, NY, United States). Cohen’s k was used to assess agreement between IOS and spirometry in the diagnosis of SAD. Continuous variables showing a normal distribution were presented as mean (standard deviation), and continuous variables without a normal distribution were presented as median [interquartile range (IQR)]. We compared baseline characteristics between discordant groups using Student’s t-test or Wilcoxon rank-sum test for normal and non-normal continuous variables, respectively, and Fisher’s exact or Chi-squared test for categorical variables and differences among four groups using Analysis of Variance (ANOVA), Kruskal–Wallis test, and chi-squared test. After adjusting for age, sex, BMI, smoking index, and smoking status, we used multivariate regression analysis to analyze factors of discordant results. Line regression adjusting baseline statistical difference variables analyzed the differences between discordant groups in CT imaging. *p* < 0.05 was considered statistically significant.

## Results

### Consistency Comparison Both the Two Sets of SAD Diagnostic Criteria

Overall, 575 SAD subjects (31.3%) were diagnosed by both spirometry and IOS; 629 subjects (34.3%) had no SAD; 435 SAD subjects (23.7%) were diagnosed by spirometry only but not IOS; 197 SAD subjects (10.7%) were diagnosed by only IOS but not spirometry. There was a slight agreement between spirometry and IOS in the assessment of SAD (kappa 0.322, *p* < 0.001). Taking spirometry as the standard, the sensitivity and specificity of IOS were 56.9% and 76.2%, respectively. The area under curve (AUC) was 0.665 (0.641–0.690) (Table 1). However, we found that in patients with normal lung function, the number of IOS-only SAD was nearly twice than that of spirometry. Similarly, we used two of the three indicators less than LLN or MMEF less than LLN as abnormal criteria to evaluate SAD. The results showed that the number of IOS-only SAD was nearly four times than that of spirometry (Supplementary Table S1). However, the number of spirometry-only SAD was more than ten times that of spirometry in patients with abnormal lung function (Supplementary Table S2).

**TABLE 1:** Comparison of diagnostic consistency between the criteria of spirometry diagnosis of SAD after bronchodilator test and impulse oscillometry diagnosis of SAD before the bronchodilator test in all subjects.

		Spirometry		Sensitive (%)	Specificity (%)	AUC (95%CI)	PPV	NPV	Kappa	*p*-Value
		Negative (-)	Positive (+)							
Impulse oscillometry	Negative (-)	629	435	56.9	76.2	0.665 (0.641 – 0.690)	74.5	59.1	0.322	<0.001
	Positive (+)	197	575								

### Clinical Characteristics of Participants in the Study

Compared with IOS-only SAD, spirometry-only SAD was older (62.34 vs. 60.23), more male (85.1 vs. 70.1%), lower body mass index (22.49 vs. 23.54), higher smoking index (28.00 vs. 6.40), more cough (24.1 vs. 13.7%), phlegm (28.5 vs. 15.2%), more drug treatment (26.7 vs. 7.1%) and family history of respiratory diseases (18.0 vs. 6.1%) (Table 2), and airflow limitation (FEV_1_/FVC) (66.37 vs. 79.34) and more severe impaired lung function (FEV_1_% predicted) (83.49 vs. 90.88) (Table 3).

**TABLE 2:** Clinical characteristics of SAD patients diagnosed by IOS and spirometry in all subjects.

	Concordant no SAD (n = 629)	Concordant SAD (n = 575)	Spirometry-only SAD (n = 435)	IOS-Only SAD (n = 197)	*p*-Value^a^
Age	57.41 (7.71)	64.75 (7.32)	62.34 (7.44)	60.23 (8.26)	0.002
Male, n (%)	331 (52.6)	521 (90.6)	370 (85.1)	138 (70.1)	<0.001
BMI, kg/m^2^	23.24 (3.16)	22.14 (3.32)	22.49 (3.09)	23.54 (3.28)	<0.001
Pack-years	0 (0–24.00)	30.00 (11.88–50.00)	28.00 (4.20–45.00)	6.40 (0–43.31)	<0.001
Smoking, n (%)					<0.001
Never	381 (60.6)	92 (16.0)	96 (22.1)	91 (46.2)	
Ever	66 (10.5)	176 (30.6)	85 (19.5)	32 (16.2)	
Current	182 (28.9)	307 (53.4)	254 (58.4)	74 (37.6)	
Clinical symptoms, n (%)					
Cough	83 (13.2)	219 (38.1)	105 (24.1)	27 (13.7)	0.003
Phlegm	104 (16.5)	241 (41.9)	124 (28.5)	30 (15.2)	<0.001
Wheeze	31 (4.9)	90 (15.7)	29 (6.7)	8 (4.1)	0.196
Dyspnea	88 (14.0)	216 (37.6)	77 (17.7)	28 (14.2)	0.270
mMRC score	0	0 (0–1)	0	0	0.208
CAT score	2 (0–5)	3 (0–7)	2 (0–5)	2 (0–5)	0.108
Family history of respiratory diseases, n (%)	46 (7.4)	92 (16.1)	78 (18.0)	12 (6.1)	<0.001
Drug treatment, n (%)	56 (8.9)	223 (38.8)	116 (26.7)	14 (7.1)	<0.001
GOLD stage, n (%)					<0.001
NO airway limitation	611 (97.1)	91 (15.8)	132 (30.3)	195 (99.0)	
GOLD 1	18 (2.9)	123 (21.4)	216 (49.7)	2 (1.0)	
GOLD 2	0 (0)	268 (46.6)	83 (19.1)	0 (0)	
GOLD 3–4	0 (0)	93 (16.2)	4 (0.9)	0 (0)	

Data are presented as the mean (standard deviation) or median (interquartile range) and were analyzed by Student’s t-test or Wilcoxon’s rank-sum test.; BMI, body mass index.

a Comparing discordant groups (spirometry-only SAD, vs. IOS-only SAD).

**TABLE 3:** Lung function features of SAD patients diagnosed by IOS and spirometry in all subjects.

	Concordant no SAD (n = 629)	Concordant SAD (n = 575)	Spirometry-only SAD (n = 435)	IOS-only SAD (n = 197)	*p*-Value
Spirometry^a^					
FEV_1_, % predicted	97.17 (11.64)	66.80 (16.92)	83.49 (12.12)	90.88 (11.25)	<0.001
FVC, % predicted	97.17 (13.73)	90.17 (16.11)	100.27 (16.05)	91.36 (13.09)	<0.001
FEV_1_/FVC	80.40 (5.71)	58.14 (11.81)	66.37 (7.57)	79.34 (5.11)	<0.001
MMEF, % predicted	100.43 (27.44)	33.44 (15.10)	46.28 (13.18)	89.86 (21.48)	<0.001
FEF_50_, % predicted	100.34 (26.04)	34.30 (17.20)	50.45 (15.89)	91.06 (21.35)	<0.001
FEF_75_, % predicted	94.79 (40.05)	31.65 (13.53)	39.09 (13.98)	84.98 (34.37)	<0.001
IOS^b^					
R_5_	0.30 (0.24–0.36)	0.40 (0.33–0.48)	0.27 (0.24–0.33)	0.36 (0.30–0.42)	<0.001
R_20_	0.27 (0.22–0.32)	0.27 (0.24–0.32)	0.25 (0.22–0.30)	0.27 (0.23–0.31)	<0.001
R_5_-R_20_	0.03 (0.01–0.05)	0.11 (0.07–0.17)	0.03 (0.01–0.04)	0.08 (0.06–0.11)	<0.001
X_5_	−0.09 (−0.11–0.07)	−0.15 (−0.21–0.11)	−0.09 (−0.11–0.07)	−0.11 (−0.14–0.09)	<0.001
AX	0.22 (0.14–0.34)	0.98 (0.57–1.85)	0.22 (0.14–0.34)	0.55 (0.38–0.84)	<0.001
F_res_	11.11 (9.23–13.44)	20.13 (16.69–23.81)	11.56 (9.45–13.89)	16.13 (14.69–18.23)	<0.001

Data are presented as the mean (standard deviation) or median (interquartile range) and were analyzed by Student’s t-test or Wilcoxon’s rank-sum test. Each oscillatory index was expressed as the mean value of three entire respiratory cycles.

a Spirometry index after the bronchodilator test.

b IOS, index before bronchodilator test; FEV_1_, forced expiratory volume in 1 s; FVC, forced vital capacity low-frequency reactance area; F_res_, resonant frequency; R_5_, Rrs at 5 Hz; R_20_, Rrs at 20 Hz; R_5_-R_20_, the difference between R_5_ and R_20_; X_5_, Xrs at 5 Hz.

### Factors Associated With Inconsistent Groups

**FIGURE 1:**
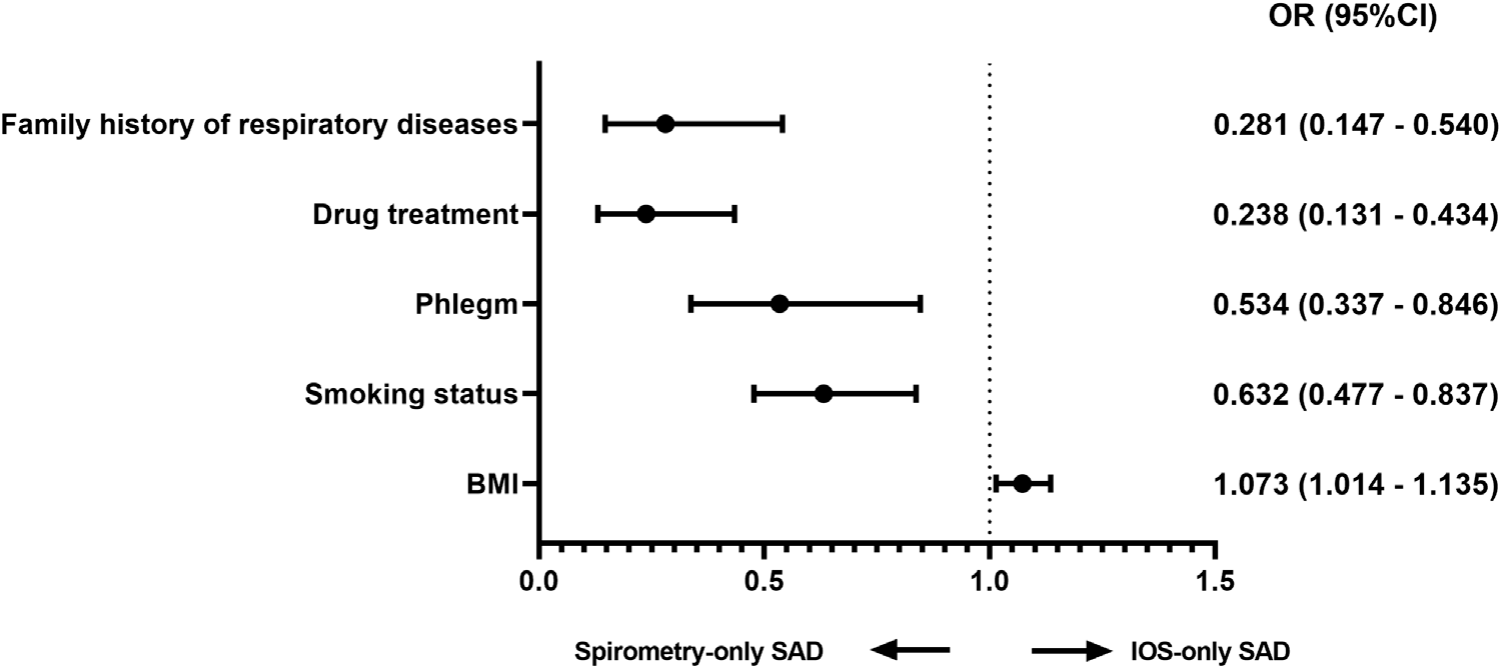
Factors associated with discordance (multivariable logistic regression) Adjusted analysis comparing IOS-only SAD and spirometry-only SAD in all subjects adjusted for age, sex, BMI, smoking status, and smoking index. Abbreviations: OR, odds ratio; BMI, body mass index.

After adjusting for age, sex, BMI, smoking index, and smoking status covariates, compared with IOS-only SAD, multivariate regression analysis showed that smoking status (OR = 0.632, 95% CI [0.477–0.837], *p* = 0.001), phlegm (0.534 95% CI [0.337–0.846], *p* = 0.008), drug treatment (0.238 95% CI [0.131–0.434], *p* < 0.001) and family history of respiratory disease (0.281 95% CI [0.147–0.540], *p* < 0.001) were factors leading to spirometry-only SAD. However, BMI (1.073 95% CI [1.014–1.135], *p* = 0.014) was the independent factor leading to IOS-only SAD (Figure 1).

### Imaging Differences Between Inconsistent Groups

**FIGURE 2:**
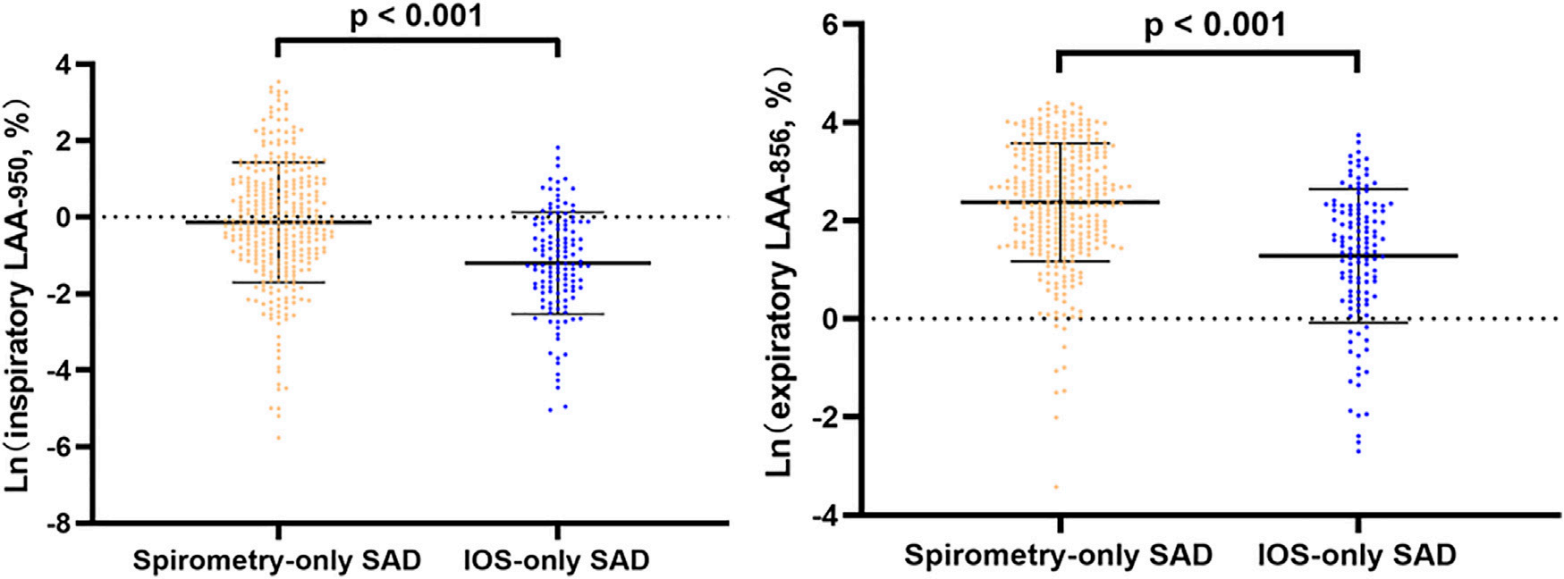
Line regression analysis of CT-emphysema and gas trapping difference between IOS-SAD and spirometry-SAD in all subjects. Adjusted for baseline statistical difference variables (age, sex, BMI, smoking status, smoking index, cough, phlegm, treatment, and family history of respiratory disorder). Ln: natural log. 342 spirometry-SAD subjects underwent CT and 129 spirometry-SAD subjects underwent CT in all subjects. Abbreviations: IOS, impulse oscillometry; SAD, small airway dysfunction; CT, computed tomography.

**FIGURE 3:**
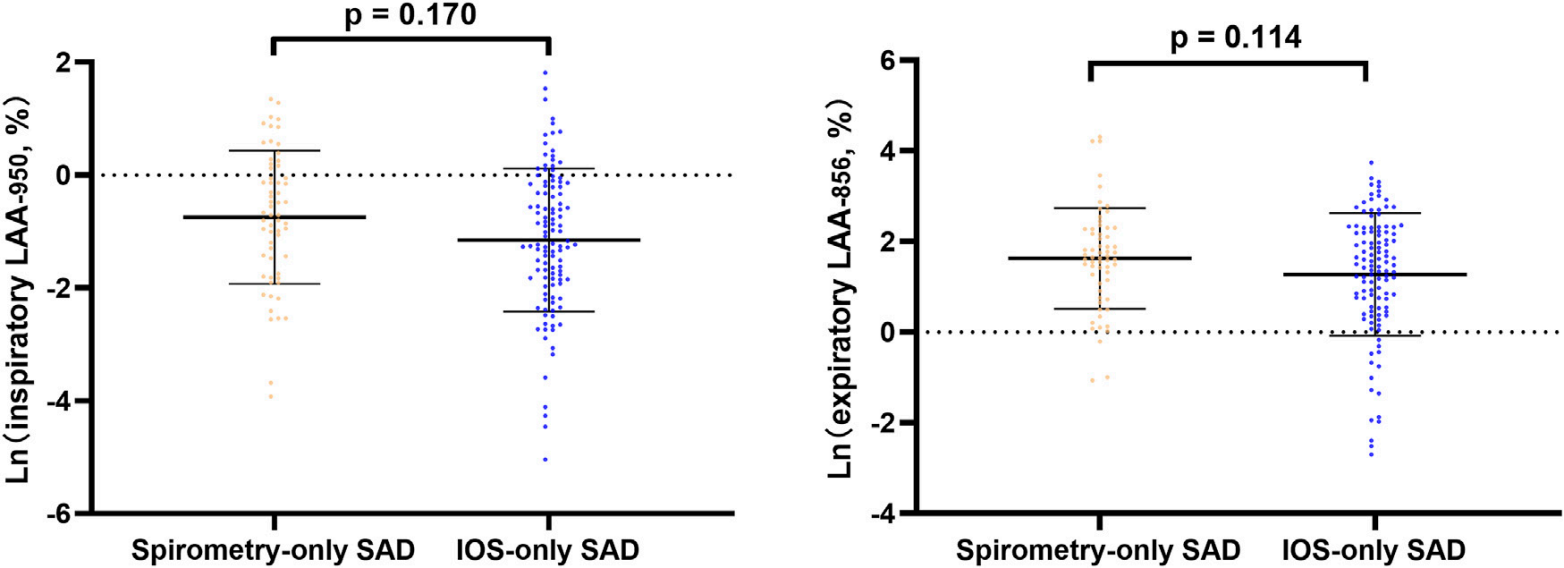
Line regression analysis of CT-emphysema and gas trapping difference between IOS-SAD and spirometry-SAD in normal lung function subjects. After adjusting for baseline statistical difference variables (age, sex, BMI, smoking status, smoking index, cough, phlegm, treatment, and family history of respiratory disorder). Ln: natural log. 61 spirometry-SAD subjects underwent CT and 113 spirometry-SAD subjects underwent CT in normal lung function subjects. Abbreviations: IOS, impulse oscillometry; SAD, small airway dysfunction; CT, computer tomography.

In line regression, after adjusting for baseline statistical difference variables, we found that in all subjects and abnormal lung function subjects, spirometry-only SAD was more severe in CT emphysema and gas trapping than IOS-only SAD (Figure 2, Supplementary Figure S1). However, in normal lung function subjects, there was no significant difference in emphysema and gas trapping between the IOS-only SAD and spirometry-only SAD groups (Figure 3).

## Discussion

This study analyzed the slight agreement between spirometry and IOS in the diagnosis of SAD. IOS may be more sensitive for evaluating SAD than spirometry in patients with normal lung function. However, in patients with abnormal lung function, spirometry may be more sensitive than IOS to detect SAD patients with clinical symptoms and CT lesions.

There was a slight consistency between spirometry and IOS in the evaluation of SAD, and we listed the following possible explanations: 1) the differences in demographic and clinical characteristics in baseline may be related to inconsistencies between the two, such as FEV_1_, age, BMI, family history of respiratory disease, smoking status, drug treatment, and phlegm. The result was similar to those reported by Brusasco et al. (2015). 2) It also may be due to the difference between respiratory patterns and detection principles (Chiu et al., 2020). From respiratory patterns, IOS applied low-amplitude pressure oscillations to the respiratory system during tidal breathing to measure respiratory impedance, whereas spirometry involved forced expiratory maneuvers that may induce airway collapse and expiratory flow limitation or complete closure (Goldman et al., 2005; Zimmermann et al., 2019). From detection principles, MMEF of spirometry was regarded as the index in the evaluation of SAD, but it was directly dependent on FVC, and the rationality of the index can be explained only when FVC is normal (Konstantinos Katsoulis et al., 2016). One study showed that there was a poor correlation between the lung function index of SAD and gas trapping (FVC and RV/TLC) (Sorkness et al., 2008), and its ability to evaluate SAD may be questioned. Compared with spirometry, the frequency dependence of R_rs_ of IOS was commonly quantified as the difference R_5_-R_20_ (Kaminsky et al., 2022). A recent study confirmed that R_5_-R_20_ was a direct indicator of anatomical narrowing in the small airways through lung computational models (Foy et al., 2019). Modeling studies suggested R_5_-R_20_ may reflect upper airway shunt flow (Thorpe et al., 2004; Bhatawadekar et al., 2015). These studies showed that the two indicators were commonly used to evaluate SAD, but they had their own shortcomings and emphasized the importance of the combination in the diagnosis of SAD.

Baseline characteristics showed that there was no significant difference in clinical symptoms between IOS-only SAD and concordant No SAD. This may indicate that airway abnormalities were detected by IOS, but it was difficult to detect by clinical symptoms and disease history. In people with normal lung function, there was no statistical difference between IOS-only SAD and spirometry-only SAD in CT imaging, which also showed that it was difficult to distinguish IOS-only SAD by imaging. We also found that most IOS-only SAD existed in people with normal lung function, and its number was about twice as much as that of spirometry-only SAD. This indicated that IOS may be more sensitive than spirometry in the assessment of SAD in normal lung function subjects, and we used different criteria and got similar results. This result was consistent with the conclusions of some studies about IOS in the assessment of SAD (Chiu et al., 2020; Li et al., 2021). This group should be worthy of our attention. We speculated that IOS-only SAD may be the early stage of abnormal development of small airways. Compared with the spirometry-only SAD, high BMI was an independent factor leading to IOS-only SAD, and there were possible reasons that obesity may cause mild inflammation and increased mechanical load on the chest, resulting in increased airway resistance (McClean et al., 2008; Umetsu, 2017). This was consistent with the results of a prospective study on the effects of BMI and pulmonary function in childhood (Ekström et al., 2018). Multivariate regression analysis showed that compared with IOS-only SAD, spirometry-only SAD subjects were older, more male, had lower body mass index, higher smoking index, more phlegm, drug treatment, and family history of respiratory diseases. From population distribution, we found most spirometry-only SAD subjects had abnormal lung function. CT imaging analysis showed that this SAD group may appear to show structural imaging changes such as emphysema and gas trapping. This indicated that spirometry-only SAD may be in the middle or late stage of abnormal development of small airways. Some studies in pathophysiology (McDonough et al., 2011; Stockley et al., 2017) showed that the severe loss of small airways occurred before emphysema. These results showed that spirometry may not detect the initial pathological abnormalities of small airways earlier, but spirometry may detect more people with clinical symptoms and abnormal CT than IOS.

This study was the first to analyze the concordance between spirometry and IOS in the assessment of SAD to analyze factors causing inconsistency and explore the applicability in different populations. Second, we also combined CT imaging to analyze the differences between discordant groups. Finally, this study was based on the Chinese IOS predicted value equation, which would be more precise to calculate IOS indexes.

There were some limitations in this study. First, this study was a cross-sectional study and had no follow-up. Second, our subjects were southern region population, and the factors of SAD may be related to air pollution and race. Finally, there were a large number of COPD patients in our baseline; however, we found that the number of IOS-only SAD was very few in COPD, and we were unable to perform a subgroup analysis of the inconsistency based on COPD severity. In addition, some studies applied other effort-independent methods (such as nitrogen washout and closing volume/capacity) to assess small airway dysfunction (Milic-Emili et al., 2007; Jetmalani et al., 2018). Although these are not used universally, there may be a missed opportunity to provide useful data on the difference between IOS and lung function.

## Conclusion

IOS may be more sensitive than spirometry in the diagnosis of SAD in normal lung function subjects. In patients with abnormal lung function, spirometry may be more sensitive than IOS to detect patients with clinical symptoms and CT lesions. The consistency of spirometry and IOS in the diagnosis of SAD highlights the necessity of combining two tools in the diagnosis of airway abnormalities. In the future, more longitudinal data would be needed to observe the progress of the discordant SAD group (developing COPD and lung function decline faster) for early intervention.

## Data Availability

The original contributions presented in the study are included in the article/Supplementary Material, further inquiries can be directed to the corresponding authors.

## Abbreviation

COPD, chronic obstructive pulmonary disease; CT, computerized tomography; FVC, forced vital capacity; FEV_1_, forced expiratory volume in one second; MMEF, maximum mid expiratory flow; IOS, impulse oscillometry; R_5_-R_20_, the difference from R_5_ to R_20_; R_5_, resistance at 5 Hz; R_20_, resistance at 20 Hz; X_5_, reactance at 5 Hz; F_res_, resonant frequency; OR, odds ratio; CI, confidence intervals; BMI, body mass index; concordant SAD, SAD assessed by both spirometry and IOS; SAD, small airway dysfunction; concordant NO SAD, SAD assessed by neither spirometry nor IOS; Spirometry-only SAD, SAD diagnosed by only spirometry but not IOS; IOS-only SAD, SAD diagnosed by only IOS but not spirometry.
