# Corrigendum: Discordant spirometry and impulse oscillometry assessments in the diagnosis of small airway dysfunction

**DOI:** 10.3389/fphys.2022.1124823

**Published:** 2023-01-10

**Authors:** Lifei Lu, Jieqi Peng, Ningning Zhao, Fan Wu, Heshen Tian, Huajing Yang, Zhishan Deng, Zihui Wang, Shan Xiao, Xiang Wen, Youlan Zheng, Cuiqiong Dai, Xiaohui Wu, Kunning Zhou, Pixin Ran, Yumin Zhou

**Affiliations:** ^1^ National Center for Respiratory Medicine, State Key Laboratory of Respiratory Disease, National Clinical Research Center for Respiratory Disease, Guangzhou Institute of Respiratory Health, The First Affiliated Hospital of Guangzhou Medical University, Guangzhou, China; ^2^ Guangzhou Laboratory, Guangzhou, China

**Keywords:** spirometry, impulse oscillometry, small airway dysfunction, COPD, computed tomography

In the published article, there was an error in Figure 1 as published, as it is not consistent with the result description**.** The corrected Figure 1 appears below:

**FIGURE 1:**
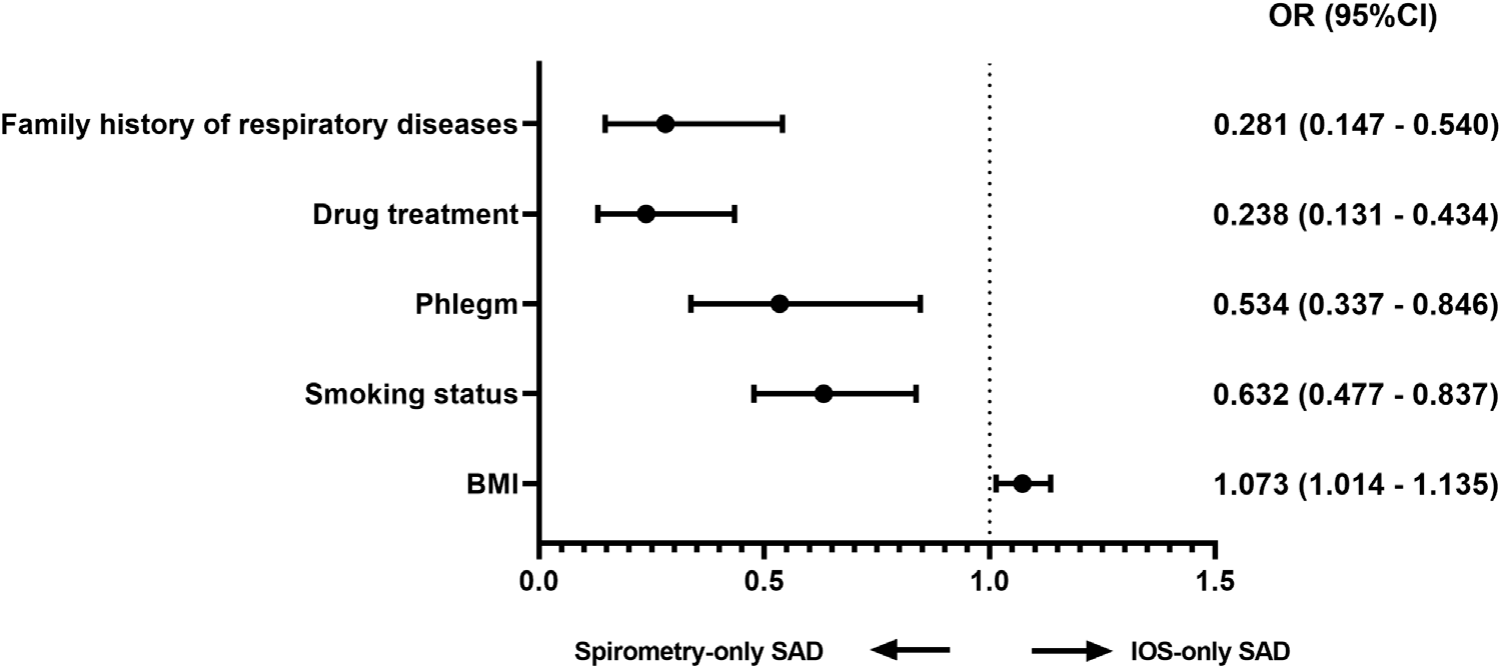
Factors associated with discordance (multivariable logistic regression) adjusted analysis comparing IOS-only SAD and spirometry-only SAD in all subjects adjusted for age, sex, BMI, smoking status, and smoking index. Abbreviations: OR, odds ratio; BMI, body mass index.

The authors apologize for this error and state that this does not change the scientific conclusions of the article in any way. The original article has been updated.

