# Supplementary material for: Discordant Spirometry and Impulse Oscillometry Assessments in the Diagnosis of Small Airway Dysfunction
Source: Front Physiol. 2022 Jun 22;13:892448. doi: 10.3389/fphys.2022.892448 (PMC9257410; doi:10.3389/fphys.2022.892448)
Supplement: Supplementary file 1 [file DataSheet1.docx]

**Supplementary Table 1** comparison of diagnostic consistency between different criteria of Spirometry diagnosis of SAD after bronchodilator test and Impulse oscillometry diagnosis of SAD (R5-R20 > ULN) before bronchodilator test in normal lung function subjects

|  |  | **Spirometry(a)** | |  | **Spirometry(b)** | |  | **Spirometry(c)** | |
| --- | --- | --- | --- | --- | --- | --- | --- | --- | --- |
|  |  | Negative (-) | Positive (+) |  | Negative (-) | Positive (+) |  | Negative (-) | Positive (+) |
| **Impulse oscillometry** | Negative (-) | 563 | 83 |  | 604 | 42 |  | 605 | 41 |
|  | Positive (+) | 164 | 33 |  | 186 | 11 |  | 188 | 9 |

Spirometry(a): at least two of the following three indicators being less than 65% of predicted: maximal mid-expiratory flow (MMEF), forced expiratory flow (FEF)50%, and FEF75% after BDT; Spirometry(b): at least two of the following three indicators being less than the limit of normal (LLN): maximal mid-expiratory flow (MMEF), forced expiratory flow (FEF)50%, and FEF75% after BDT; Spirometry(c): maximal mid-expiratory flow (MMEF) less than the limit of normal (LLN) after BDT

**Supplementary Table 2** comparison of diagnostic consistency between different criteria of clinical Spirometry diagnosis of SAD after bronchodilator test and Impulse oscillometry diagnosis of SAD (R5-R20 > ULN) before bronchodilator test in abnormal lung function subjects

|  |  | **Spirometry(a)** | |  | **Spirometry(b)** | |  | **Spirometry(c)** | |
| --- | --- | --- | --- | --- | --- | --- | --- | --- | --- |
|  |  | Negative (-) | Positive (+) |  | Negative (-) | Positive (+) |  | Negative (-) | Positive (+) |
| **Impulse oscillometry** | Negative (-) | 66 | 352 |  | 126 | 292 |  | 123 | 295 |
|  | Positive (+) | 33 | 542 |  | 60 | 515 |  | 60 | 515 |

Spirometry(a): at least two of the following three indicators being less than 65% of predicted: maximal mid-expiratory flow (MMEF), forced expiratory flow (FEF)50%, and FEF75% after BDT; Spirometry(b): at least two of the following three indicators being less than the limit of normal (LLN): maximal mid-expiratory flow (MMEF), forced expiratory flow (FEF)50%, and FEF75% after BDT; Spirometry(c): maximal mid-expiratory flow (MMEF) less than the limit of normal (LLN) after BDT


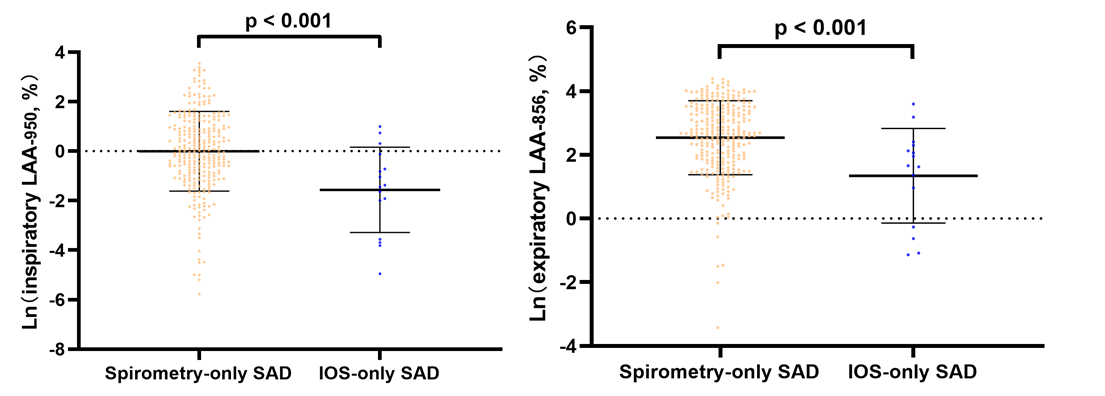


**Supplementary Figure 1** Line regression analyze CT-emphysema and gas trapping difference between IOS-SAD and Spirometry-SAD in abnormal lung function subjects. Adjusted for baseline statistical difference variables (age, sex, BMI, smoking status, smoking index, cough, phlegm, treatment, family history of respiratory). Ln:natural log, 281 spirometry-SAD subjects underwent CT and 16 spirometry-SAD subjects underwent CT in abnormal lung function subjects.
